# Liver metastasis affects progression pattern during immune checkpoint inhibitors monotherapy in gastric cancer

**DOI:** 10.3389/fonc.2023.1193533

**Published:** 2023-09-15

**Authors:** Iori Motoo, Takayuki Ando, Takeru Hamashima, Shinya Kajiura, Miho Sakumura, Yuko Ueda, Aiko Murayama, Kohei Ogawa, Kenichiro Tsukada, Akira Ueda, Nobuhiro Suzuki, Naokatsu Nakada, Koji Nakashima, Ayumu Hosokawa, Ichiro Yasuda

**Affiliations:** ^1^ Third Department of Internal Medicine, University of Toyama, Toyama, Japan; ^2^ Department of Pathology, University of Toyama, Toyama, Japan; ^3^ Department of Gastroenterology, Toyama Prefectural Central Hospital, Toyama, Japan; ^4^ Department of Gastroenterology, Kouseiren Takaoka Hospital, Takaoka, Japan; ^5^ Department of Medical Oncology, Toyama Red Cross Hospital, Toyama, Japan; ^6^ Department of Gastroenterology, Jouetsu Sogo Hospital, Jouetsu, Japan; ^7^ Department of Gastroenterology, Itoigawa Sogo Hospital, Itoigawa, Japan; ^8^ Department of Clinical Oncology, University of Miyazaki Hospital, Miyazaki, Japan

**Keywords:** gastric cancer, immune checkpoint inhibitor, progression pattern, liver metastasis, tumor-infiltrating lymphocytes

## Abstract

**Introduction:**

The efficacy of immune checkpoint inhibitors (ICIs) is heterogeneous at each metastatic site, and tumor progression pattern is associated with survival; however, it remains unclear in gastric cancer (GC). Therefore, we aimed to clarify the progression pattern in response to ICIs in patients with GC, and we analyzed its mechanism focusing on the intratumoral immune cells.

**Methods:**

Patients who received ICIs were retrospectively classified into non-systemic and systemic progression groups based on their radiological assessments. Moreover, the best percentage change in target lesions from each organ was compared.

**Results:**

Among 148 patients, the non-systemic progression group showed a significant improvement in overall survival (OS) compared with the systemic progression group (median, 5.6 months vs. 3.3 months; HR, 0.53; 95%CI, 0.32–0.89; p = 0.012). Poor performance status (HR, 1.73, 95%CI, 1.00–2.87) and systemic progression (HR, 3.09, 95%CI, 1.95–4.82) were associated with OS. Of all metastatic sites, the liver showed the poorest percentage change, and liver metastasis (OR, 2.99, 95%CI, 1.04–8.58) was associated with systemic progression. Hence, intratumoral CD8+ T-cell density was lower in patients with liver metastasis than in those without liver metastasis after ICIs, although the density of CD4+ T-cells (Th1, Th17, and Treg) and CD163+ cells (TAM) were not significantly different.

**Conclusion:**

The new progression pattern was associated with OS in GC. Liver metastasis may be a predictive factor of systemic progression during ICIs by regulating intratumoral CD8+ T-cells.

## 1 Introduction

Gastric cancer (GC) is the fifth most common cancer and the fourth leading cause of cancer-related mortality in the world (1). Although fluoropyrimidines and platinum-based chemotherapy as first-line therapy and taxanes plus ramucirumab as second-line therapy are standard treatment for GC, prognosis remains poor and median survival is approximately one year (2–5).

Recently, immune checkpoint inhibitors (ICIs), such as anti-programmed cell death-1 (PD-1) or programmed cell death ligand-1 (PD-L1) monoclonal antibodies, have improved overall survival (OS) for various cancers including GC (6–10). Nivolumab and pembrolizumab, a monoclonal antibody against PD-1, were approved for GC in Japan. The pivotal phase III trial (ATTRACTION-2) demonstrated that nivolumab in the third- or later-line treatment for GC exhibited a survival benefit in the Asian population (11). Two phase III trials, ATTRACTION-4 and Checkmate-649, have recently shown that nivolumab has promising activity in combination with chemotherapy as first-line treatment in patients with GC (12, 13). Pembrolizumab has also been approved as a second-line treatment for patients with microsatellite instability-high (MSI-H) or mismatch repair deficient (MMR-D) solid tumors, including GC, by the US Food and Drug Administration (14).

Anti-PD-1 antibodies exhibit promising activity by restoring an efficient antitumor T-cell response within the tumor microenvironment. Therefore, the presence of tumor-infiltrating lymphocytes (TILs) and PD-L1 expression in tumors are predictive factors associated with the ICI response (15–17). However, the subtypes of TILs vary based on the metastatic organ site because of tissue-specific immunoregulation (18). However, the other components of the tumor microenvironment, such as tumor-associated macrophages (TAM) and dendritic cells, affect the response to ICI treatment (19). These findings suggest that the response to ICI treatment is different at each metastatic site and depends on the tumor-immune microenvironment.

A recent study indicated that the response to ICIs was different at each metastatic site and the progression pattern was heterogeneous in non-small cell lung cancer (NSCLC) and MMR-D tumors. Lymph nodes had a significantly better response than lung, pleural, and liver lesions in NSCLC and MMR-D tumors (20), and liver lesions exhibited the poorest response to PD-1 blockade (17, 20). Surprisingly, recent studies have revealed liver immune tolerance, and they suggest that liver metastases can be used to determine immunotherapy efficacy by affecting systemic antitumor activity, including the regulation of CD8+ T-cells and CD4+ T-cells (21–23). Moreover, the liver is a common site of GC metastases, and liver metastasis has been reported to be a risk factor associated with hyperprogressive disease (HPD) in cases of GC treated with ICIs (24).

ICIs may cause a rapid proliferation known as HPD (25). HPD was defined as a more than two-fold increase in the tumor growth rate (TGR) compared with that at the evaluation of disease progression during the previous line of treatment (26). A previous study reported that HPD was associated with poor survival in patients with NSCLC treated with PD-1/PD-L1 inhibitors (27). However, in GCs, there was no difference in survival time between the HPD groups and PD without HPD groups (28). Thus, a new progression pattern has been required as a prognostic factor in GCs.

Currently, the response at metastatic sites, the progression pattern, and liver immune tolerance in GC patients treated with ICIs remain unclear. Therefore, in this study, we examined the response to ICIs at each individual metastatic site, the association of progression pattern with survival, and intratumoral immune cells by liver metastasis status in patients with GC receiving ICIs.

## 2 Materials and methods

### 2.1 Patients

Patients were enrolled with unresectable or recurrent GC who had received at least one cycle of nivolumab or pembrolizumab monotherapy after failing prior treatment that included fluoropyrimidine plus platinum-based, taxane-based, trifluridine/tipiracil, or irinotecan chemotherapy. All patients were treated between October 2017 and December 2020 at nine institutions, including the Toyama University Hospital. We reviewed medical records, which included gender, age, Eastern Cooperative Oncology Group (ECOG) performance status (PS), histologic type, HER2 status, MSI status, history of gastrectomy, metastatic site, number of metastatic sites, history of chemotherapy before nivolumab or pembrolizumab monotherapy, and laboratory assessments. Patients received nivolumab (240 mg/body biweekly) until disease progression, the occurrence of unacceptable toxicity, or patient refusal to continue therapy. Patients with MSI-high GC received pembrolizumab (200 mg/body triweekly). This study was approved by the institutional review boards of each participating institute including the Toyama University Hospital (ethic code: R2021016).

### 2.2 Radiologic assessments and predefined progression pattern

Computed tomography (CT) scans of the patients during ICI treatment were reviewed. The objective response of the measurable lesions was assessed according to Response Evaluation Criteria in Solid Tumor version 1.1 (RECIST v1.1). Patients without measurable lesions were excluded from the response rate analysis. The absolute size and percent change of individual metastases were quantified using unidimensional measurements (millimeters). Nontarget and new lesions were captured and followed qualitatively. The level of ascites was classified into no, mild (limited to the pelvic cavity or around the liver), moderate (neither mild nor massive), and massive (continuous ascites from the liver surface to the pelvic cavity) using CT. The best ascites response was determined in patients who initially had ascites as follows: complete response (CR), the disappearance of ascites; partial response (PR), the decrease in severity of ascites by at least one level as described above; stable disease (SD), other than CR, PR, or progressive disease (PD); PD, increase by at least one level; not evaluated, ascites drained during treatment; or no CT (29).

HPD was defined as the tumor growth kinetics ratio (TGK_R_) of more than two and more than a 50% increase in tumor burden compared with that at pre-treatment imaging, based on a previous study (26). The time of pre-baseline, baseline, and post-CT scanning was defined as *T_pre_*, *T_0_*, and *T_post_*, respectively. The sum of the largest diameters of the target lesions according to RECIST v1.1 at pre-baseline, baseline, and post-CT was defined as *S_pre_*, *S_0_*, and *S_post_*, respectively. TGK_pre_ was calculated as the difference in the sum of the largest diameters of the target lesions per unit time between pre-baseline and baseline imaging: (*S*_0–_ *S_pre_*)/(*T_0–_T_pre_*). Similarly, TGK_post_ was calculated as follows: (*S*_post–_ *S0*)/(*T_post–_T_0_*). We defined TGK_post_/TGK_pre_ as TGK_R_.

Based on the best response to ICIs by RECIST v1.1, the progression pattern was defined as follows: systemic progression, PD in two or more organs, including target, nontarget, or new lesions developed in different organs; and non-systemic progression, PD in only one organ, including target, nontarget, or new lesions based on the criteria of a previous study (20).

### 2.3 Immunohistochemistry

Slides were stained with hematoxylin and eosin stain as well as with anti-CD8 (Nichirei, Tokyo, Japan), anti-CD4 (Nichirei, Tokyo, Japan), anti-FOXP3 (Abcam, Cambridge, MA), and anti-CD163 (Leica Biosystems, Nussloch, Germany) antibodies at the Toyama University Hospital. Additionally, slides were double-stained either with anti-CD4 (Proteintech, Chicago, IL) and anti-T-bet (Cell Signaling Danvers, MA) antibodies or with anti-CD4 and anti-IL17 (Proteintech, Chicago, IL) antibodies. All stained slides were evaluated by one pathologist and one investigator who were trained to identify the features of GC. As described previously, the slides were examined for the presence of CD8+ T-cells, CD4+ T-cells (Th1, Th17, and Treg), and CD163+ cells at the invasive tumor margin (30). The images were acquired using an OLYMPUS BX61 microscope (Olympus, Tokyo, Japan) at 20× and 40× magnifications. We counted the number of CD8+ T-cells, CD4+ T-cells (Th1, Th17, and Treg), and CD163+ cells within the tumor margin of three independent fields at 40× magnification and calculated the density of the CD8+ T-cells, CD4+ T-cells (Th1, Th17, and Treg), and CD163+ cells (cells/mm^2^) (17, 30).

### 2.4 Statistical analysis

Progression-free survival (PFS) was defined as the start of PD-1 therapy until RECIST-defined PD or death or, if no progression, was censored at the data lock. OS was defined as the start of PD-1 therapy until death or, if no progression, was censored at the data lock among groups that were depicted using the Kaplan–Meier method and compared using the log-rank test. Categorical variables were calculated as frequencies and percentages and continuous variables as mean and range. They were assessed using Fisher’s exact test, chi-square test, Student’s t-test, or the Mann–Whitney U-test. The clinical factors that were significantly associated with OS or systemic progression in the univariate analyses were further assessed in multivariate Cox proportional hazard model analyses.

A *p-value* less than 0.05 was considered statistically significant. Statistical analyses were performed using JMP, version 15.0 (SAS Institute, Cary, NC) and GraphPad Prism 7.0 software (GraphPad, La Jolla, CA).

## 3 Results

### 3.1 Patient characteristics

A total of 148 patients treated with nivolumab or pembrolizumab for GC as salvage therapy were included in this study. The baseline characteristics are shown in **Table 1**. The median age was 70 (range, 36–90). A large percentage of the patients were male (65%), ECOG PS of 0–1 (72%), and the majority had two or more metastatic sites (60%) and ascites (59%). The common metastatic sites were lymph nodes (59%), followed by the liver (47%), peritoneum (52%), and lung (8%). Of 28 patients evaluated with the MSI assay, four exhibited MSI-high status, and all patients received pembrolizumab as second-line treatment.

**Table 1:** Baseline characteristics of patients treated with PD-1 blockade.

Characteristics	Number of patients (n = 148)
Gender	
Male	96
Female	52
Age	
Median (range)	70 (36–90)
15–< 65	40
65–< 75	66
≥75	42
ECOG performance status	
0	8
1	99
≥2	41
Primary site	
Gastric	139
Esophagogastric junction	9
Histopathologic type	
Intestinal	71
Diffuse	77
HER2 status	
Positive	31
Negative	117
MSI status/MMR deficiency	
Positive	4
Negative	24
Not evaluate	120
NLR	
<3	86
≥3	62
Stage	
Advanced	114
Postoperative recurrence	34
Metastatic sites	
Lymph node	88
Liver	70
Peritoneum	78
Lung	12
Bone	8
Ascites	
Mild	45
Moderate	12
Massive	31
Number of metastatic sites	
1	58
≥2	90
Treatment line before ICI	
1	5
2	106
3	22
≥4	15
Previous treatments	
Fluoropyrimidine*	147
Platinum**	129
Taxan***	134
Irinotecan	20
Trifluridine-tipiracil	5
Trastuzumab	26
Ramucirumab	118
Others	1
ICI drugs	
Nivolumab	144
Pembrolizumab	4

MSI, microsatellite instability; MMR, mismatch repair; NLR, neutrophil-to-lymphocyte ratio; ICI, immune checkpoint inhibitor.

*Including 5-FU, S-1 and capecitabine, **Including cisplatin and oxaliplatin, ***Including paclitaxel and docetaxel.

### 3.2 Response and survival

According to RECIST v1.1, the objective response rate (ORR) was 11.6% (13/112) and disease control rate (DCR) was 41.0% (46/112) in patients with measurable lesions (**Table 2**). The median PFS and OS were 1.6 (95%CI, 1.4–1.9) months and 4.4 (95%CI, 3.6–6.6) months, respectively (**Supplementary Figures 1A, B**).

**Table 2:** Response to PD-1 blockade.

	Number of patients (n = 148)
Target lesion	112
CR	2
PR	11
SD	13
PD	57
Not evaluated	9
RR (%)	11.6
DCR (%)	41

CR, Complete response; PR, partial response; SD, stable disease; PD, progressive disease; RR, response rate; DCR, disease control rate.

**Figure 1:**
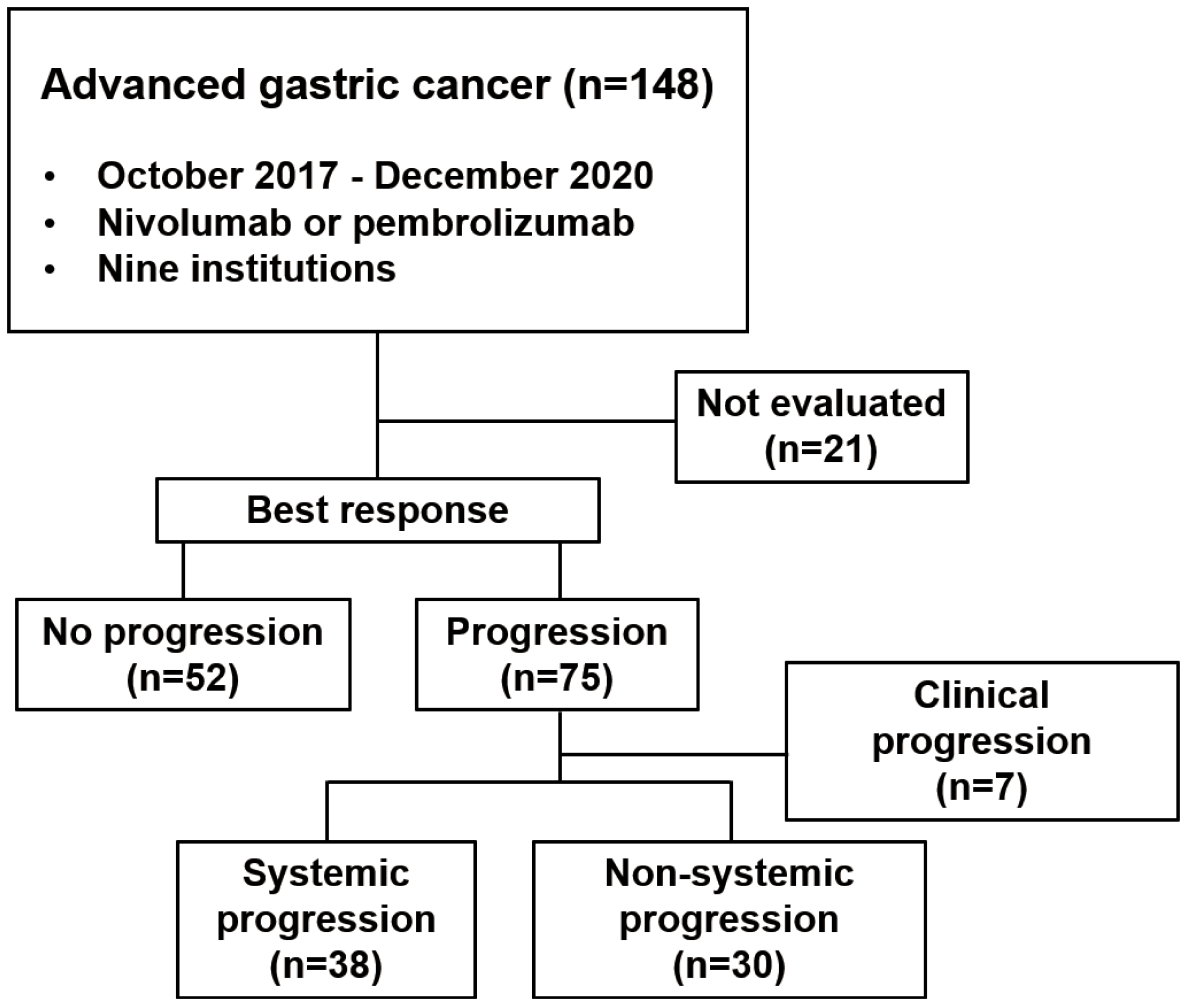
Flowchart of the study process.

**Figure 2:**
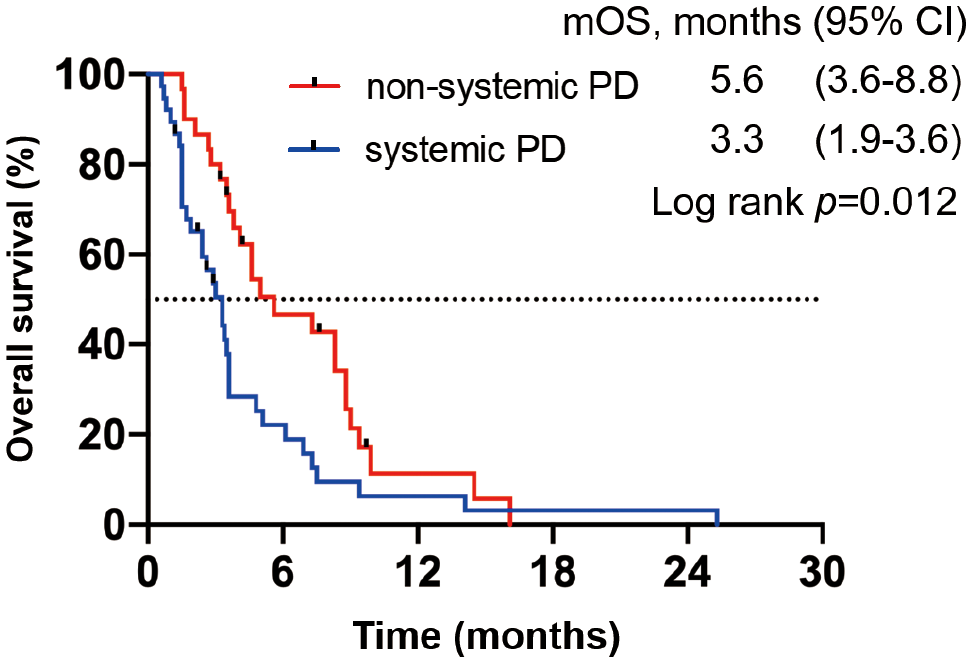
OS in patients from the progression group based on the best response to ICIs. Kaplan–Meier OS curve; patients with systemic progression (n = 38) are shown in blue, whereas those with non-systemic progression (n = 30) are shown in red.

We divided all patients into progression and no progression groups based on the best response to ICI therapy. Among 75 patients in the progression group, seven were excluded from the analysis because of clinical progression without radiological progression. Thus, 68 patients were eligible for inclusion and were divided into systemic progression and non-systemic progression groups. Finally, 38 (56%) patients belonging to the systemic progression group and 30 (44%) patients belonging to the non-systemic progression group (**Figure 1**) were evaluable. Subsequently, we determined the association between the progression pattern and OS. As expected, patients with non-systemic progression had a significantly longer OS than those with systemic progression (median, 5.6 months vs. 3.3 months; hazard ratio [HR], 0.53; 95% confidence interval [95%CI], 0.32–0.89; p = 0.012; **Figure 2**).

### 3.3 Univariate and multivariate analyses of prognostic factors associated with OS

To confirm that the progression pattern was an independent prognostic factor for GC patients treated with ICIs, we examined the prognostic factors associated with OS in all patients. In a univariate analysis of PS (0–1 or ≥2), resection of the primary site (yes or no), number of metastatic sites (0–1 or ≥2), liver metastasis (yes or no), peritoneal metastasis (yes or no), Neutrophil-to-Lymphocyte Ratio (NLR) (<3 or ≥3), HPD (yes or no), and progression pattern (systemic progression or non-systemic progression) as covariates, PS (HR, 2.58; 95%CI, 1.70–3.85; p < 0.0001), NLR (HR, 1.61; 95%CI, 1.11–2.32; p = 0.019) and progression pattern (HR, 3.34; 95%CI, 2.12–5.17; p < 0.0001) were significantly associated with OS. In a multivariate analysis, poor PS (HR, 1.73; 95%CI, 1.00–2.87; p = 0.049) and systemic progression (HR, 3.09; 95%CI, 1.95–4.82; p < 0.0001) were significantly associated with OS (**Table 3**).

**Table 3:** Univariate and multivariate analyses of factors associated with overall survival.

Factor	Univariate analysis			Multivariate analysis		
	HR	95%CI	p	HR	95%CI	p
Performance status, 0-1 (/≥2)	2.58	1.70–3.85	<0.0001	1.73	1.00–2.87	0.049
Resection of primary sites, yes (/no)	0.96	0.65–1.41	0.87			
Numbers of metastatic sites, 0–1 (/≥2)	0.95	0.66–1.39	0.80			
Liver metastasis, yes (/no)	1.36	0.94–1.96	0.09			
Peritoneal metastasis, yes (/no)	1.42	0.99–2.06	0.055			
NLR, <3 (/≥3)	1.61	1.11–2.32	0.019	1.11	0.71–1.72	0.63
Hyperprogressive disease, yes (/no)	1.36	0.68–2.44	0.35			
Progression pattern, systemic progression (/non-systemic progression)	3.34	2.12–5.17	<0.0001	3.09	1.95–4.82	<0.0001

NLR, neutrophil-to-lymphocyte ratio.

### 3.4 Univariate and multivariate analyses of clinical features associated with systemic progression

To explore the predictive factors of systemic progression, we examined clinical features associated with systemic progression using baseline clinical characteristics (**Supplementary Table 1**). The proportion of patients with liver metastases and NLR ≥ 3 was significantly larger in the systemic progression group than in the non-systemic progression group. In a univariate analysis of PS (0–1 or ≥2), resection of primary site (yes or no), number of metastatic sites (0–1 or ≥2), liver metastasis (yes or no), peritoneal metastasis (yes or no), NLR (<3 or ≥3), and HPD (yes or no) as covariates, only liver metastasis (OR, 3.68; 95%CI, 1.33–10.1; p = 0.011) and NLR (OR, 3.05; 95%CI, 1.09–8.55; p = 0.033) were associated with systemic progression. In a multivariate analysis, the presence of liver metastasis (OR, 2.99; 95%CI, 1.04–8.58; p = 0.04) was an independent factor of systemic progression (**Table 4**).

**Table 4:** Univariate and multivariate analyses of factors associated with systemic progression.

Factor	Univariate analysis			Multivariate analysis		
	OR	95%CI	p	OR	95%CI	p
Performance status, 0–1 (/≥2)	1.12	0.38–3.26	0.83			
Resection of primary sites, yes (/no)	2.45	0.90–6.68	0.079			
Numbers of metastatic sites, 0–1 (/≥2)	2.45	0.88–6.77	0.08			
Liver metastasis, yes (/no)	3.68	1.33–10.1	0.011	2.99	1.04–8.58	0.04
Peritoneal metastasis, yes (/no)	1.85	0.70–4.89	0.21			
NLR, <3 (/≥3)	3.05	1.09–8.55	0.033	2.32	0.71–1.72	0.12
Hyperprogressive disease, yes (/no)	1.73	0.46–6.42	0.41			

NLR, neutrophil-to-lymphocyte ratio.

### 3.5 The site of metastasis and response to ICI treatment

**Figure 3:**
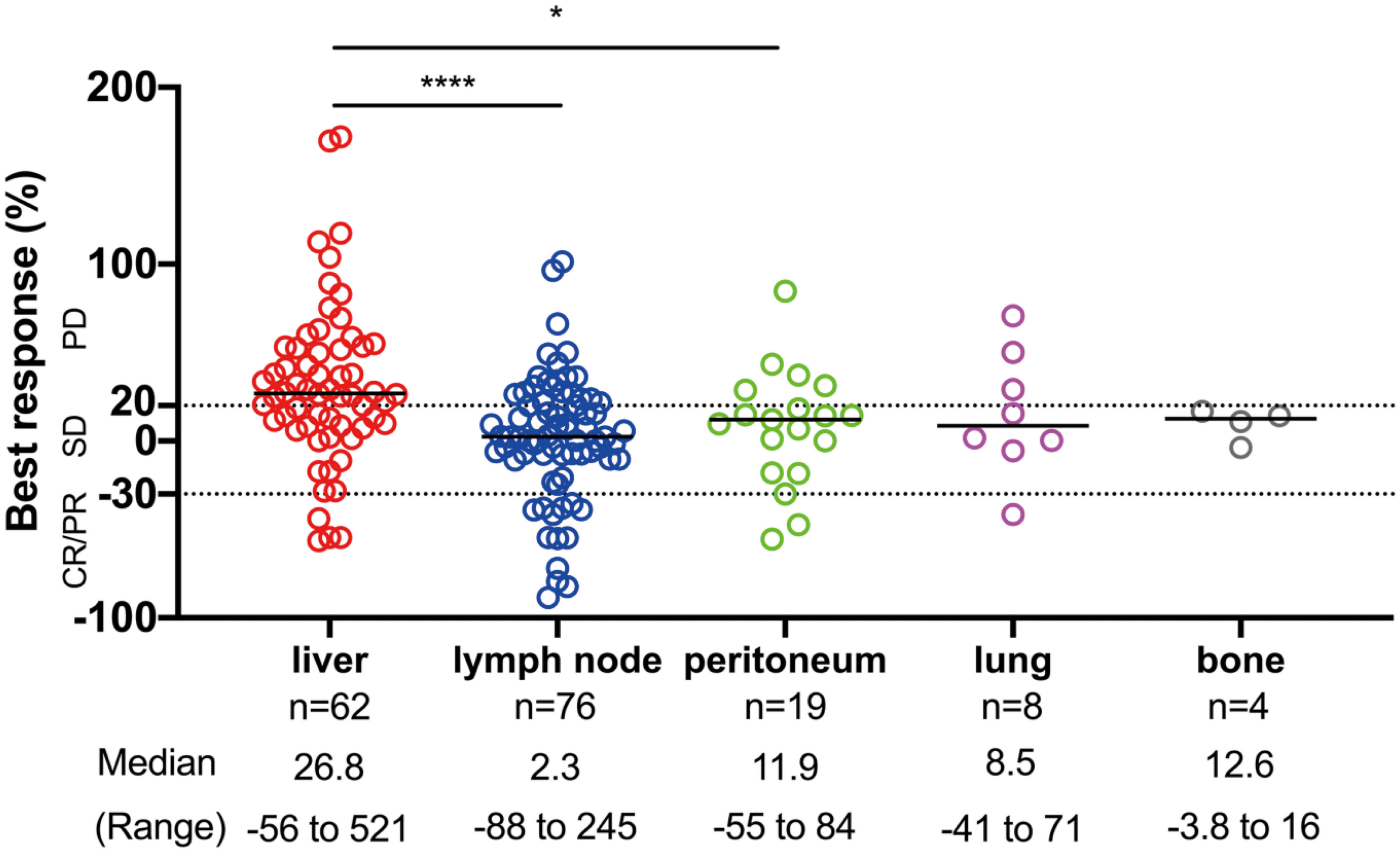
The site of disease and response to ICI therapy. The distribution of the best response (%) of individual target lesions to ICIs by the organ site of metastasis. The response by RECIST criteria, the median, and the range of percentage of responses are depicted. Comparisons among responses: *p < 0.05, ****p < 0.0001. CR, complete response; PR, partial response; SD, stable disease; PD, progressive disease.

We considered that the difference in progression pattern is associated with the difference in response to ICI therapy for each organ because the progression pattern of ICIs is associated with survival and liver metastasis is a predictive factor associated with systemic progression. Therefore, we examined whether the organ site of the metastasis was associated with response to PD-1 blockade in patients with measurable lesions. Individual metastases tended to have the best responses in lymph nodes; followed by the peritoneum, lung, and bone with intermediate responses, whereas the liver exhibited the poorest responses (**Figure 3**). Liver lesions had significantly poorer responses than lymph node and peritoneal lesions (p < 0.0001, and p = 0.016, respectively). These results demonstrated that the efficacy of ICIs was heterogeneous within each organ. Additionally, the tumor response was not related to tumor size in the liver or lymph node metastases (**Supplementary Figure 2**).

### 3.6 Tumor margin CD8+ T-cell count before and after ICI treatment

**Figure 4:**
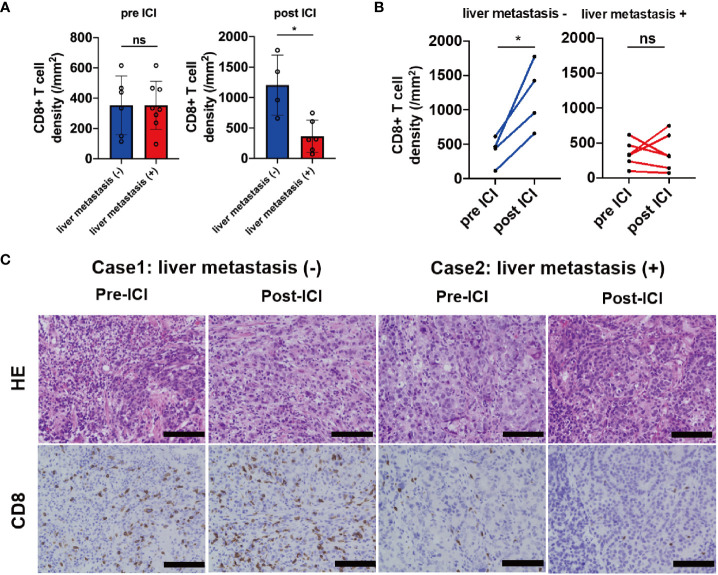
IHC analysis of CD8 in samples obtained from patients before and after the ICI treatment. **(A)** CD8+ T-cell densities before (liver metastasis (+) group n = 8; liver metastasis (–) group n = 6) and after (liver metastasis (+) group n = 6; liver metastasis (–) group n = 4) ICI based on the liver metastasis status, **(B)** the kinetics of CD8+ T-cell densities based on the liver metastasis status (liver metastasis (+) group n = 6; liver metastasis (–) group n = 4), **(C)** representative cases of CD8 expression in samples obtained from the primary site based on the liver metastasis status. Magnification, ×20. Black bar = 100 µm. *p < 0.05; ns, not significant.

We investigated the association between tumor-associated T-cell infiltrates and liver metastasis using quantitative IHC analysis of CD8 expression at invasive margin in biopsy samples obtained from the primary site before (n = 14) and after (n = 10) ICI treatment. In the samples before the ICI treatment, the CD8+ T-cell density at the invasive margin showed no significant difference between the liver metastasis (+) and liver metastasis (−) group. However, CD8+ T-cell density was significantly lower in the liver metastasis (+) group than in the liver metastasis (−) group after the ICI treatment (p = 0.019) (**Figure 4A**). Compared with pre-ICI CD8+ T-cell density, post-ICI CD8+ T-cell density significantly increased in the liver metastasis (−) group (n = 4) but not in the liver metastasis (+) group (n = 6) (**Figure 4B**). In fact, the CD8+ T-cell density increased after ICI treatment in Case 1 without liver metastasis; however, it did not increase in Case 2 with liver metastasis (**Figure 4C**). In contrast, the densities of CD4+ T-cells (Th1, Th17, and Treg) and CD163+ cells (TAM) were not significantly different between the two groups before and after the ICI treatment (**Supplementary Figures 3A, B**). These results suggest that liver metastasis regulates CD8+ T-cell infiltration into other organs. Furthermore, PFS was significantly shorter in patients with liver metastasis compared with those without liver metastasis (median 1.4 months vs. 1.8 months, HR, 0.68; 95%CI, 0.48–0.96, p = 0.019; **Supplementary Figure 4**). ORR was also significantly lower in the liver metastasis (+) group compared with the liver metastasis (−) group (5.7% vs. 20.9%, p = 0.03; **Supplementary Table 2**). Additionally, we evaluated the clinical factors associated with CD8 increase; diffuse type and absence of liver metastasis tended to be correlated with CD8 increase (**Supplementary Table 3**).

## 4 Discussion

This study revealed that systemic progression is an independent prognostic factor in patients with GCs who received ICI monotherapy. In addition, liver metastasis was statistically associated with systemic progression and had a significantly poorer response to PD-1 blockade than metastatic lesions in other organs. These findings indicate that liver metastasis is an independent factor for the reduced efficacy observed in the ICI treatment of GC.

Recent studies reported that patients with EGFR/ALK/ROS1 mutation, bone metastasis, and no immune-related adverse events had poor prognosis during immunotherapy in lung cancer (31–33). These factors might affect the tumor immune environment, such as decreased CD8+ T-cell infiltration (34), increased Th17 cells and lack of Th1 cells in bone marrow (35), respectively. In our study, patients with poor PS and systemic progression exhibited poor OS in a multivariate analysis. Furthermore, patients with systemic progression had reduced OS compared with patients with mixed progression in NSCLC and MMR-D patients (19). The pattern of disease progression during ICI monotherapy has been associated with poor prognosis in GC (36). We demonstrated that a completely different progression pattern was an independent prognostic factor in GC treated with ICIs. The subgroup analysis in the DELIVER trial indicated that peritoneal metastasis and poor PS were negatively associated with OS and PFS (28). Another multicenter retrospective study concluded that liver metastasis, peritoneal metastasis, CRP more than 1.0 mg/dL, PD-L1 CPS more than ten, and MMR proficiency were independent prognostic factors for PFS during nivolumab treatment (37).

HPD was reported as a progression pattern for ICI treatment and defined as a rapid proliferation with an incidence of 6%–29% in various cancers (24, 38). HPD occurred in 20.5% of GCs treated with nivolumab (28). Although the mechanism of HPD is unclear, upregulation of alternative immune checkpoints resulting in further immune suppression, a low number of senescent CD4+ T-cells, and an increase of regulatory T-cells with proliferative capacity in the TIL population may cause rapid disease progression through a PD-1 blockade (39–41). The current definition of HPD does not consider the progression of nontarget lesions and the occurrence of new lesions because only the sum of the target lesions is used, based on RECIST v1.1 (38). As a result, HPD may not reflect the acceleration of tumor disease in gastrointestinal cancers with nontarget lesions including the primary site and ascites. New lesions in different organs and appearance/increase of ascites are associated with poor prognosis in AGC patients during nivolumab monotherapy (36). Therefore, we defined a new progression pattern including the progression of nontarget lesions and new lesions. Furthermore, we indicated that the systemic progression was significantly associated with poor OS in AGC patients receiving ICI monotherapy.

Liver metastasis has been suggested to decrease the probability of a response to ICI treatment by liver-induced immune tolerance (17, 42). Hence, liver metastases are associated with a reduced response and PFS in patients with melanoma and NSCLC treated with ICIs (17). Indeed, a recent meta-analysis reported that ICI- combined chemotherapy did not show survival benefit compared with platinum-based chemotherapy in NSCLC patients with liver metastasis (43). Therefore, liver metastasis should be considered a predictor of poor prognosis in lung cancer patients receiving ICI treatment (44). However, the association between liver metastasis and the efficacy of ICI remains unclear in gastric cancer. We demonstrated that liver metastasis was a predictive factor associated with systemic progression, and ORR and PFS after ICI treatment were poorer in GC patients with liver metastasis than in those without liver metastasis. Regarding its mechanism, recent experiments have demonstrated that liver metastases siphon activated CD8+ T-cells from the systemic circulation by promoting antigen-specific T-cell apoptosis within the liver in rodent models (23). Additionally, the presence of liver metastases was associated with fewer CD8+ T-cell infiltration at the cutaneous metastasis in patients with melanoma (17). These findings suggest that liver metastasis may lead to systemic loss of T-cells and diminished the efficacy of immunotherapy (45). Thus, we analyzed the number of CD8+ T-cells at the primary site before and after ICI treatment to evaluate this hypothesis. The primary site was one of the most suitable lesions for this analysis because we could evaluate the efficacy of ICI treatment and obtain biopsy samples using esophagogastroduodenoscopy. The findings in this study appear consistent with this hypothesis, although the sample size was small for immunohistochemistry. Additionally, we examined other immune-related cells in the tumor microenvironment, including CD4+ T-cells (Th1, Th17, and Treg) and CD163+ cells. We found that CD4+ T-cell (Th1, Th17, and Treg) and CD163+ cell densities were not significantly different between the two groups before and after the ICI treatment. These results suggest that liver metastasis specifically affects CD8+ T-cell infiltration in the tumor margin after the ICI treatment. Moreover, other mechanisms of liver-induced systemic immune tolerance have been reported, including the incomplete activation of CD8+ T-cells (46, 47), poor CD4+ T-cell activation (47), and activation of regulatory T-cells (46). Furthermore, tumor neoantigens and antigen-presenting cells (APCs) in the tumor microenvironment have been reported to be correlated with CD8+ T-cell infiltration (48). In this study, we confirmed that there was no difference in CD163+ cell density between the two groups. However, we did not evaluate other APCs, including dendritic cells in tumors. Thus, other components of APCs might affect CD8+ T-cell infiltration at the primary site.

In our study, the presence of liver metastases could be a potential negative baseline factor for ICI monotherapy. Therefore, the management of liver metastases might improve the efficacy of immunotherapy. Interestingly, liver-mediated radiotherapy reshapes the liver’s immune microenvironment and reduces hepatic siphoning of T-cells (23). Gamma knife radiosurgery and intensity-modulated radiotherapy have been shown to improve survival and response rates in patients with hepatocellular carcinoma (49, 50). Other approaches for liver metastases include surgical resection, radiofrequency ablation, and transarterial chemoembolization, but it is unclear whether these therapies improve hepatic immune tolerance. Therefore, further studies on other systemic therapies for hepatic tolerance are needed. In recent studies, vascular endothelial growth factor modulates the functions of immune cells toward immunosuppression (51). Moreover, vascular-normalizing therapies such as endocrine therapy and cyclin-dependent kinase 4 and 6 inhibitors can reprogram the immunosuppressive tumor microenvironment (52, 53). These findings suggest that anti-angiogenetic therapy and vascular normalization approaches might be a strategy for restoring the efficacy of immunotherapy in GC patients with liver metastases.

This study had several limitations. First, selection bias cannot be eliminated because of the retrospective study. Second, our predefined “systemic progression” and “non-systemic progression” criteria were not generally confirmed. Therefore, future studies in large cohorts are needed to confirm and modify these progression patterns. Third, we did not evaluate immune-related cell density in the metastatic organs, including the liver, peritoneum, and lungs. This is because that we could not obtain those samples in clinical situations. Alternatively, we examined readily available biopsy samples from the primary site. Despite providing support for previous studies, our immunohistochemical study was insufficient. Fourth, although our study confirmed that the progression pattern was associated with the survival outcome of ICI treatment including nivolumab and pembrolizumab in GC, the findings could be influenced by different ICIs. Finally, the sample size was small for immunohistochemistry. A larger sample size is required to determine the association of immune-related cells in the tumor microenvironment with liver metastasis; however, CD8+ T-cell density increased after ICI treatment in all four cases without liver metastasis. We hope that more prospective clinical research will be conducted in the future to confirm our conclusions.

In conclusion, our study demonstrated that the new progression pattern was associated with the survival outcome of ICI treatment in GC. Furthermore, liver metastasis may be a predictive factor of systemic progression during ICI treatment by regulating CD8+ T-cell infiltration into tumors. These results suggest that the management of liver metastases might improve immunotherapy efficacy and provide a different perspective for future trials.

## Data availability statement

The data presented in this study are available on request from the corresponding author.

## Ethics statement

The studies involving humans were approved by Toyama University Hospital Institutional Review Board. The studies were conducted in accordance with the local legislation and institutional requirements. The ethics committee/institutional review board waived the requirement of written informed consent for participation from the participants or the participants’ legal guardians/next of kin because the retrospective nature of the study.

## Author contributions

Conception and design: IM, TA, and IY; collection and assembly of data: IM, TA, SK, MS, YU, AM, KO, KT, AU, NS, NN, KN, and AH; data analysis and interpretation: IM, TA, and TH; Manuscript writing: IM and TA. All authors contributed to the article and approved the submitted version.

## References

[1] Sung H Ferlay J Siegel RL Laversanne M Soerjomataram I Jemal A. Global Cancer Statistics 2020: GLOBOCAN estimates of incidence and mortality worldwide for 36 cancers in 185 countries. C.A. Cancer J Clin (2021) 71:209–49. doi: 10.3322/caac.21660 33538338

[2] Koizumi W Narahara H Hara T Takagane A Akiya T Takagi M. S-1 plus cisplatin versus S-1 alone for first-line treatment of advanced gastric cancer (SPIRITS trial): a phase III trial. Lancet Oncol (2008) 9:215–21. doi: 10.1016/S1470-2045(08)70035-4 18282805

[3] Yamada Y Higuchi K Nishikawa K Gotoh M Fuse N Sugimoto N. Phase III study comparing oxaliplatin plus S-1 with cisplatin plus S-1 in chemotherapy-naive patients with advanced gastric cancer. Ann Oncol (2015) 26:141–8. doi: 10.1093/annonc/mdu472 25316259

[4] Wilke H Muro K Van Cutsem E Oh SC Bodoky G Shimada Y. Ramucirumab plus paclitaxel versus placebo plus paclitaxel in patients with previously treated advanced gastric or gastro-oesophageal junction adenocarcinoma (RAINBOW): a double-blind, randomised phase 3 trial. Lancet Oncol (2014) 15:1224–35. doi: 10.1016/S1470-2045(14)70420-6 25240821

[5] Fuchs CS Tomasek J Yong CJ Dumitru F Passalacqua R Goswami C. Ramucirumab monotherapy for previously treated advanced gastric or gastro-oesophageal junction adenocarcinoma (REGARD): an international, randomised, multicentre, placebo-controlled, phase 3 trial. Lancet (2014) 383:31–9. doi: 10.1016/S0140-6736(13)61719-5 24094768

[6] Robert C Long GV Brady B Dutriaux C Maio M Mortier L. Nivolumab in previously untreated melanoma without BRAF mutation. N Engl J Med (2015) 372:320–30. doi: 10.1056/NEJMoa1412082 25399552

[7] Borghaei H Paz-Ares L Horn L Spigel DR Steins M Ready NE. Nivolumab versus docetaxel in advanced nonsquamous non-small-cell lung cancer. N Engl J Med (2015) 373:1627–39. doi: 10.1056/NEJMoa1507643 PMC5705936 26412456

[8] Motzer RJ Escudier B McDermott DF George S Hammers HJ Srinivas S. Nivolumab versus everolimus in advanced renal-cell carcinoma. N Engl J Med (2015) 373:1803–13. doi: 10.1056/NEJMoa1510665 PMC5719487 26406148

[9] Ferris RL Blumenschein GJ Fayette J Guigay J Colevas AD Licitra L. Nivolumab for recurrent squamous-cell carcinoma of the head and neck. N Engl J Med (2016) 375:1856–67. doi: 10.1056/NEJMoa1602252 PMC5564292 27718784

[10] Rittmeyer A Barlesi F Waterkamp D Park K Ciardiello F von Pawel J. Atezolizumab versus docetaxel in patients with previously treated non-small-cell lung cancer (OAK): a phase 3, open-label, multicentre randomised controlled trial. Lancet (2017) 389:255–65. doi: 10.1016/S0140-6736(16)32517-X PMC6886121 27979383

[11] Kang YK Boku N Satoh T Ryu MH Chao Y Kato K. Nivolumab in patients with advanced gastric or gastro-oesophageal junction cancer refractory to, or intolerant of, at least two previous chemotherapy regimens (ONO-4538-12, ATTRACTION-2): a randomised, double-blind, placebo-controlled, phase 3 trial. Lancet (2017) 390:2461–71. doi: 10.1016/S0140-6736(17)31827-5 28993052

[12] Kang YK Chen LT Ryu MH Oh DY Oh SC Chung HC. Nivolumab plus chemotherapy versus placebo plus chemotherapy in patients with HER2-negative, untreated, unresectable advanced or recurrent gastric or gastro-oesophageal junction cancer (ATTRACTION-4): a randomised, multicentre, double-blind, placebo-controlled, phase 3 trial. Lancet Oncol (2022) 23:234–47. doi: 10.1016/S1470-2045(21)00692-6 35030335

[13] Janjigian YY Shitara K Moehler M Garrido M Salman P Shen L. First-line nivolumab plus chemotherapy versus chemotherapy alone for advanced gastric, gastro-oesophageal junction, and oesophageal adenocarcinoma (CheckMate 649): a randomised, open-label, phase 3 trial. Lancet (2021) 398:27–40. doi: 10.1016/S0140-6736(21)00797-2 34102137 PMC8436782

[14] Marabelle A Le DT Ascierto PA Di Giacomo AM De Jesus-Acosta A Delord JP. Efficacy of pembrolizumab in patients with noncolorectal high microsatellite instability/mismatch repair-deficient cancer: results from the phase II KEYNOTE-158 Study. J Clin Oncol (2020) 38:1–10. doi: 10.1200/JCO.19.02105 31682550 PMC8184060

[15] Herbst RS Soria JC Kowanetz M Fine GD Hamid O Gordon MS. Predictive correlates of response to the anti-PD-L1 antibody MPDL3280A in cancer patients. Nature (2014) 515:563–7. doi: 10.1038/nature14011 PMC4836193 25428504

[16] Topalian SL Hodi FS Brahmer JR Gettinger SN Smith DC McDermott DF. Safety, activity, and immune correlates of anti-PD-1 antibody in cancer. N Engl J Med (2012) 366:2443–54. doi: 10.1056/NEJMoa1200690 PMC3544539 22658127

[17] Tumeh PC Hellmann MD Hamid O Tsai KK Loo KL Gubens MA. Liver metastasis and treatment outcome with anti-PD-1 monoclonal antibody in patients with melanoma and NSCLC. Cancer Immunol Res (2017) 5:417–24. doi: 10.1158/2326-6066.CIR-16-0325 PMC5749922 28411193

[18] Pao W Ooi CH Birzele F Ruefli-Brasse A Cannarile MA Reis B. Tissue-specific immunoregulation: a call for better understanding of the "Immunostat" in the context of cancer. Cancer Discov (2018) 8:395–402. doi: 10.1158/2159-8290.CD-17-1320 29545369

[19] Teng MW Ngiow SF Ribas A Smyth MJ. Classifying cancers based on T-cell infiltration and PD-L1. Cancer Res (2015) 75:2139–45. doi: 10.1158/0008-5472.CAN-15-0255 PMC4452411 25977340

[20] Osorio JC Arbour KC Le DT Durham JN Plodkowski AJ Halpenny DF. Lesion-level response dynamics to programmed cell death protein (PD-1) blockade. J Clin Oncol (2019) 37:3546–55. doi: 10.1200/JCO.19.00709 PMC7194449 31675272

[21] Crispe IN. Hepatic T cells and liver tolerance. Nat Rev Immunol (2003) 3:51–62. doi: 10.1038/nri981 12511875

[22] Bamboat ZM Stableford JA Plitas G Burt BM Nguyen HM Welles AP. Human liver dendritic cells promote T cell hyporesponsiveness. J Immunol (2009) 182:1901–11. doi: 10.4049/jimmunol.0803404 PMC3254024 19201843

[23] Yu J Green MD Li S Sun Y Journey SN Choi JE. Liver metastasis restrains immunotherapy efficacy via macrophage-mediated T cell elimination. Nat Med (2021) 27:152–64. doi: 10.1038/s41591-020-1131-x PMC8095049 33398162

[24] Liu J Wu Q Wu S Xie X. Investigation on potential biomarkers of hyperprogressive disease (HPD) triggered by immune checkpoint inhibitors (ICIs). Clin Transl Oncol (2021) 23:1782–93. doi: 10.1007/s12094-021-02579-9 33847923

[25] Champiat S Dercle L Ammari S Massard C Hollebecque A Postel-Vinay S. Heyperprogressive disease is a new pattern of progression in cancer patients treated by anti-PD-1/PD-L1. Clin Cancer Res (2017) 23:1920–8. doi: 10.1158/1078-0432.CCR-16-1741 27827313

[26] Sasaki A Nakamura Y Mishima S Kawazoe A Kuboki Y Bando H. Predictive factors for hyperprogressive disease during nivolumab as anti-PD1 treatment in patients with advanced gastric cancer. Gastric Cancer (2019) 22:793–802. doi: 10.1007/s10120-018-00922-8 30627987

[27] Ferrara R Mezquita L Texier M Lahmar J Audigier-Valette C Tessonnier L. Hyperprogressive disease in patients with advanced non-small cell lung cancer treated with PD-1/PD-L1 inhibitors or with single-agent chemotherapy. JAMA Oncol (2018) 4:1543–52. doi: 10.1001/jamaoncol.2018.3676 PMC6248085 30193240

[28] Takahashi Y Sunakawa Y Inoue E Kawabata R Ishiguro A Kito Y. Real-world effectiveness of nivolumab in advanced gastric cancer: the DELIVER trial (JACCRO GC-08). Gastric Cancer (2022) 25:235–44. doi: 10.1007/s10120-021-01237-x 34427838

[29] Nakajima TE Yamaguchi K Boku N Hyodo I Mizusawa J Hara H. Randomized phase II/III study of 5-fluorouracil/l-leucovorin versus 5-fluorouracil/l-leucovorin plus paclitaxel administered to patients with severe peritoneal metastases of gastric cancer (JCOG1108/WJOG7312G). Gastric Cancer (2020) 23:677–88. doi: 10.1007/s10120-020-01043-x 32036492

[30] Tumeh PC Harview CL Yearley JH Shintaku IP Taylor EJ Robert L. PD-1 blockade induces responses by inhibiting adaptive immune resistance. Nature (2014) 515:568–71. doi: 10.1038/nature13954 PMC4246418 25428505

[31] Zhou J Lu X Zhu H Ding N Zhang Y Xu X. Resistance to immune checkpoint inhibitors in advanced lung cancer: Clinical characteristics, potential prognostic factors and next strategy. Front Immunol (2023) 14:1089026. doi: 10.3389/fimmu.2023.1089026 36776868 PMC9910216

[32] Shankar B Zhang J Naqash AR Forde PM Feliciano JL Marrone KA. Multisystem immune-related adverse events associated with immune checkpoint inhibitors for treatment of non-small cell lung cancer. JAMA Oncol (2020) 6:1952–56. doi: 10.1001/jamaoncol.2020.5012 PMC7596677 33119034

[33] Su X Roudi R Dai T Chen S Fan B Li H. Immune-related adverse events associated with programmed cell death protein-1 and programmed cell death ligand 1 inhibitors for non-small cell lung cancer: a PRISMA systematic review and meta-analysis. BMC Cancer (2019) 19:588. doi: 10.1158/1078-0432.CCR-15-3101 31182061 PMC6558759

[34] Gainor JF Shaw AT Sequist LV Fu X Azzoli CG Piotrowska Z. EGFR mutations and ALK rearrangements are associated with low response rates to PD-1 pathway blockade in non-small cell lung cancer: a retrospective analysis. Clin Cancer Res (2016) 22:4585–93. doi: 10.1158/1078-0432.CCR-15-3101 PMC5026567 27225694

[35] Li X Wang L Chen S Zhou F Zhao J Zhao W. Adverse impact of bone metastases on clinical outcomes of patients with advanced non-small cell lung cancer treated with immune checkpoint inhibitors. Thoracic cancer (2020) 11:2812–19. doi: 10.1111/1759-7714.13597 PMC7529562 32779372

[36] Aoki M Kadowaki S Takahashi N Suzuki T Oshima K Ando T. Pattern of disease progression during third-line or later chemotherapy with nivolumab associated with poor prognosis in advanced gastric cancer: a multicenter retrospective study in Japan. Gastric Cancer (2023) 26:132–44. doi: 10.1007/s10120-022-01349-y PMC9813080 36316527

[37] Hagi T Kurokawa Y Kawabata R Omori T Matsuyama J Fujitani K. Multicentre biomarker cohort study on the efficacy of nivolumab treatment for gastric cancer. Br J Cancer (2020) 123:965–72. doi: 10.1038/s41416-020-0975-7 PMC7492241 32616848

[38] Park HJ Kim KW Won SE Yoon S Chae YK Tirumani SH. Definition, incidence, and challenges for assessment of hyperprogressive disease during cancer treatment with immune checkpoint inhibitors: a systematic review and meta-analysis. JAMA Netw Open (2021) 4:e211136. doi: 10.1001/jamanetworkopen.2021.1136 33760090 PMC7991969

[39] Koyama S Akbay EA Li YY Herter-Sprie GS Buczkowski KA Richards WG. Adaptive resistance to therapeutic PD-1 blockade is associated with upregulation of alternative immune checkpoints. Nat Commun (2016) 7:10501. doi: 10.1038/ncomms10501 26883990 PMC4757784

[40] Lo Russo G Moro M Sommariva M Cancila V Boeri M Centonze G. Antibody-Fc/FcR interaction on macrophages as a mechanism for hyperprogressive disease in non-small cell lung cancer subsequent to PD-1/PD-L1 blockade. Clin Cancer Res (2019) 25:989–99. doi: 10.1158/1078-0432.CCR-18-1390 30206165

[41] Kamada T Togashi Y Tay C Ha D Sasaki A Nakamura Y. PD-1+ regulatory T cells amplified by PD-1 blockade promote hyperprogression of cancer. Proc Natl Acad Sci USA (2019) 116:9999–10008. doi: 10.1073/pnas.1822001116 31028147 PMC6525547

[42] Funazo T Nomizo T Kim YH. Liver metastasis is associated with poor progression-free survival in patients with non-small cell lung cancer treated with nivolumab. J Thorac Oncol (2017) 12:e140–1. doi: 10.1016/j.jtho.2017.04.027 28838713

[43] Petrelli F Ferrara R Signorelli D Ghidini A Proto C Roudi R. Immune checkpoint inhibitors and chemotherapy in first-line NSCLC: a meta-analysis. Immunotherapy (2021) 13:621–31. doi: 10.2217/imt-2020-0224 33775103

[44] Xia H Zhang W Zhang Y Shang X Liu Y Wang X. Liver metastases and the efficacy of immune checkpoint inhibitors in advanced lung cancer: A systematic review and meta-analysis. Front Oncol (2022) 12:978069. doi: 10.3389/fonc.2022.978069 36330494 PMC9623244

[45] Lee JC Green MD Huppert LA Chow C Pierce RH Daud AI. The liver-immunity nexus and cancer immunotherapy. Clin Cancer Res (2022) 28:5–12. doi: 10.1158/1078-0432.CCR-21-1193 34285059 PMC8897983

[46] Jenne CN Kubes P. Immune surveillance by the liver. Nat Immunol (2013) 14:996–1006. doi: 10.1038/ni.2691 24048121

[47] Limmer A Ohl J Kurts C Ljunggren HG Reiss Y Groettrup M. Efficient presentation of exogenous antigen by liver endothelial cells to CD8+ T cells results in antigen-specific T-cell tolerance. Nat Med (2000) 6:1348–54. doi: 10.1038/82161 11100119

[48] Wang JCE Livingstone AM. Cutting edge: CD4+ T cell help can be essential for primary CD8+ T cell responses in vivo. J Immunol (2003) 171:6339–43. doi: 10.4049/jimmunol.171.12.6339 14662830

[49] Su K Guo T Xu K Wang J Liao H Li X. Gamma knife radiosurgery versus transcatheter arterial chemoembolization for hepatocellular carcinoma with portal vein tumor thrombus: propensity score matching study. Hepatol Int (2022) 16:858–67. doi: 10.1007/s12072-022-10339-2 PMC9349123 35729469

[50] Su K Guo L Ma W Wang J Xie Y Rao M. PD-1 inhibitors plus anti-angiogenic therapy with or without intensity modulated radiotherapy for advanced hepatocellular carcinoma: A propensity score matching study. Front Immunol (2022) 13:972503. doi: 10.3389/fimmu.2022.972503 36211350 PMC9539675

[51] Fukumura D Kloepper J Amoozgar Z Duda DG Jain RK. Enhancing cancer immunotherapy using antiangiogenics: opportunities and challenges. Nat Rev Clin Oncol (2018) 15:325–40. doi: 10.1038/nrclinonc.2018.29 PMC5921900 29508855

[52] Jain RK Safabakhsh N Sckell A Keshet E. Endothelial cell death, angiogenesis, and microvascular function after castration in an androgen-dependent tumor: Role of vascular endothelial growth factor. Proc Natl Acad Sci USA (1998) 95:10820–25. doi: 10.1073/pnas.95.18.10820 PMC27979 9724788

[53] Goel S DeCristo MJ Watt AC BrinJones H Sceneay J Li BB. CDK4/6 inhibition triggers anti-tumor immunity. Nature (2017) 548:471–5. doi: 10.1038/nature23465 PMC5570667 28813415

