# Supplementary material for: Liver metastasis affects progression pattern during immune checkpoint inhibitors monotherapy in gastric cancer
Source: Front Oncol. 2023 Sep 15;13:1193533. doi: 10.3389/fonc.2023.1193533 (PMC10542891; doi:10.3389/fonc.2023.1193533)
Supplement: Supplementary file 1 [file DataSheet_1.pdf]

Supplementary Material

Liver Metastasis Affects Progression Pattern During Immune Checkpoint Inhibitors Monotherapy in Gastric Cancer

Iori Motoo[1], Takayuki Ando[1], Takeru Hamashima[2], Shinya Kajiura[1], Miho Sakumura[1], Yuko Ueda[1], Aiko Murayama[1], Kohei Ogawa[3], Kenichiro Tsukada[4], Akira Ueda[5], Nobuhiro Suzuki[6], Naokatsu Nakada[7], Koji Nakashima[8], Ayumu Hosokawa[1, 8], Ichiro Yasuda[1]

*** Correspondence: Takayuki Ando**:takayuki@med.u-toyama.ac.jp

1 Supplementary Figures and Tables

1.1 Supplementary Figures

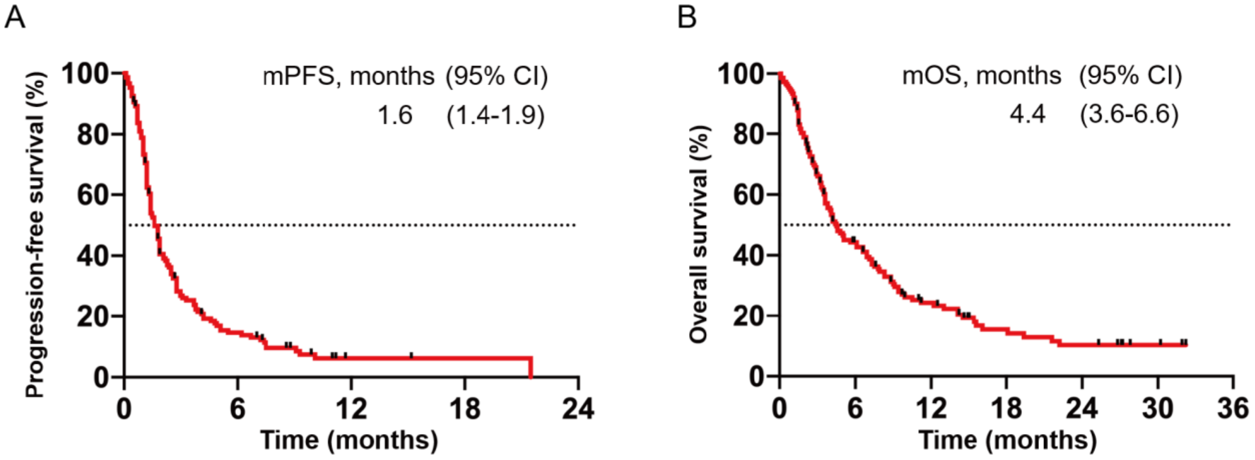

Supplementary Figure 1. PFS and OS of all patients with GC treated with ICIs. (A) Kaplan–Meier PFS curve. (B) Kaplan–Meier OS curve.

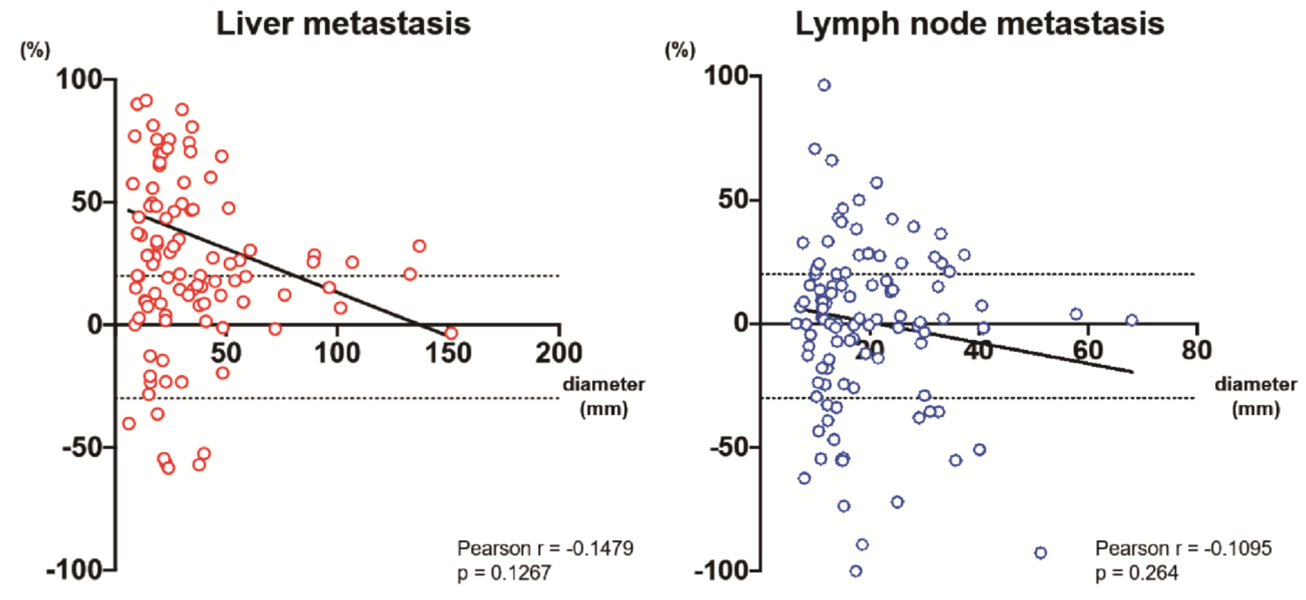

Supplementary Figure 2. The correlation between tumor response and tumor size in liver and lymph node metastasis.

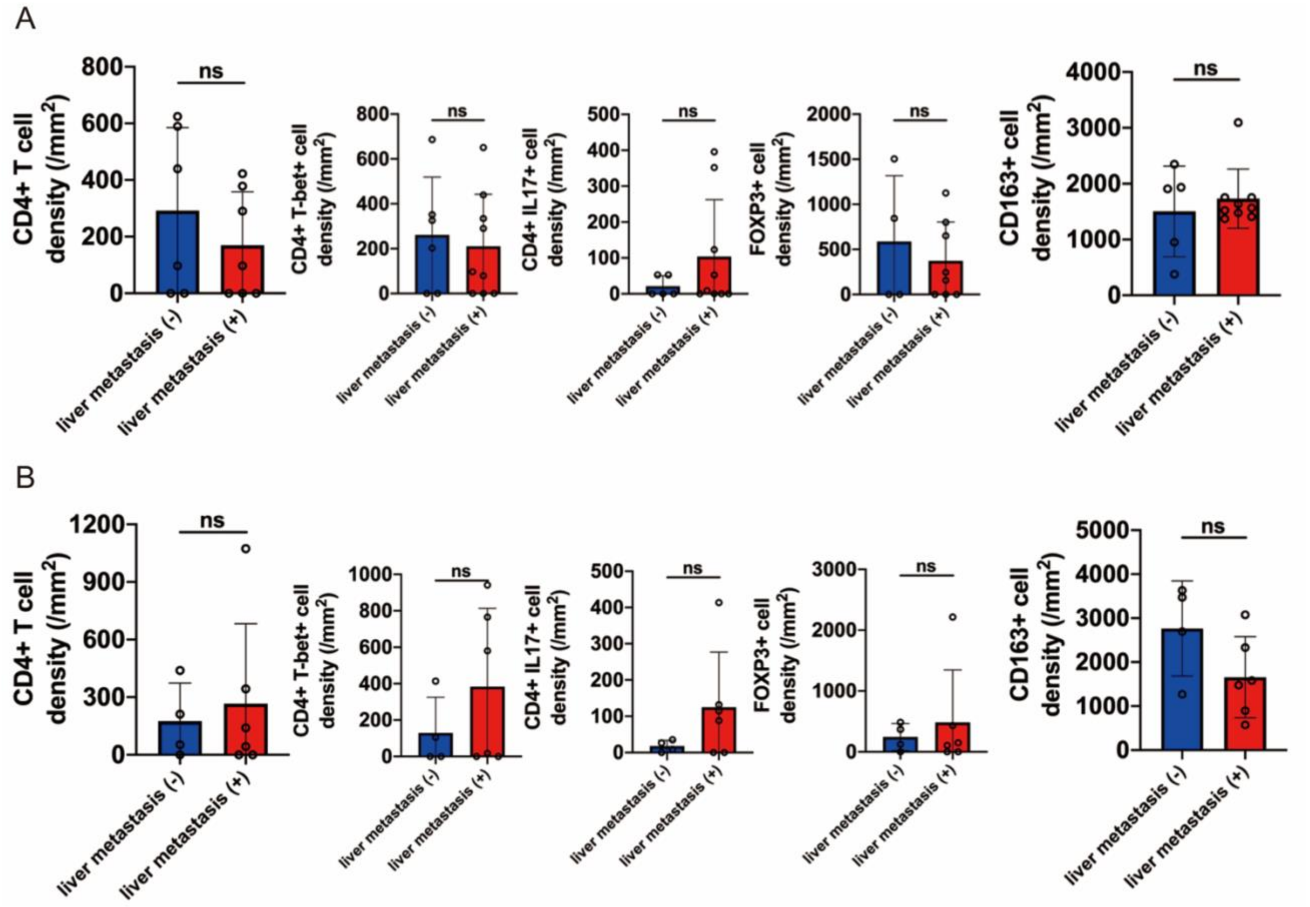

Supplementary Figure 3. IHC analysis of Th1, Th17, Treg, and TAM in samples obtained from patients before and after the ICI treatment based on the liver metastasis status. (A) CD4+ T-bet+ cell, CD4+ IL17+ cell, FOXP3+ cell, and CD163+ cell densities based on the liver metastasis status before the ICI treatment (liver metastasis (+) group n = 9; liver metastasis (–) group n = 6), (B) CD4+ T-bet+ cell, CD4+ IL17+ cell, FOXP3+ cell, and CD163+ cell densities based on the liver metastasis status after the ICI treatment (liver metastasis (+) group n = 6; liver metastasis (–) group n = 4). ns, not significant.

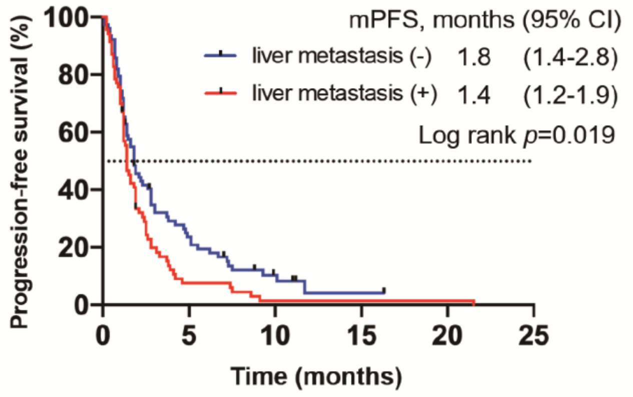

Supplementary Figure 4. PFS in all patients. Kaplan–Meier PFS curve; patients with liver metastasis (n = 70) are shown in red, whereas those without liver metastasis (n = 78) are shown in blue.

1.2 Supplementary Tables

Supplementary Table 1. Baseline characteristics of patients treated with PD-1 blockade in the progression group.

Characteristics	Systemic progression (n = 38)	Non-systemic progression (n = 30)	p value
Gender			
Male	26	21	0.88
Female	12	9	
Age Median (range)	70 (39–90)	71 (47–82)	0.68
ECOG performance status			
0–1	27	22	0.83
≥2	11	8	
Histopathologic type			
Intestinal	21	12	0.21
Diffuse	17	18	
HER2 status			
Positive	9	5	0.55
Negative	29	25	
NLR			
<3	18	22	0.03
≥3	20	8	
Stage			
Advanced	11	15	0.076
Postoperative recurrence	27	15	

Metastatic sites			
Lymph node	23	17	0.74
Liver	27	12	0.01
Peritoneum	21	12	0.17
Number of metastatic sites			
1	10	14	0.082
≥2	28	16	
PD organs			
Target lesion	33	15	0.0013
Nontarget lesion	27	12	0.01
New lesion	7	3	0.49
Hyperprogressive disease	8	4	0.52

NLR, neutrophil-lymphocyte ratio; PD, progressive disease.

Supplementary Table 2. Response to PD-1 blockade in the liver metastasis and non-liver metastasis groups.

	Number of patients	
	Liver metastasis (+)	Liver metastasis (−)
Target lesion	69	43
CR	1	1
PR	3	8
SD	20	13
PD	39	18
Not evaluated	6	3
RR (%)	5.7	20.9
DCR (%)	34.7	51.1

CR, Complete response; PR, partial response; SD, stable disease; PD, progressive disease; RR, response rate; DCR, disease control rate.

Supplementary Table 3. Baseline characteristics of patients treated with PD-1 blockade based on CD8 status.

Characteristics	CD8 increase (n = 6)	CD8 decrease (n = 4)	p value
Gender			
Male	4	4	0.46
Female	2	0	
Age<br>Median (range)	68 (54–86)	69 (65–74)	0.92
ECOG performance status			
0–1	4	4	>0.99
≥2	2	0	
Histopathologic type			
Intestinal	2	4	0.072
Diffuse	4	0	
HER2 status			
Positive	2	1	>0.99
Negative	4	3	
MSI status			
Positive	2	0	0.50
Negative	4	3	
Not evaluate	0	1	
NLR			0.50
<3	1	2	

≥3	5	2	
Metastatic sites			
Lymph node	2	3	0.52
Liver	2	4	0.072
Peritoneum	5	4	>0.99
Number of metastatic sites			
1	2	0	0.46
≥2	4	4	
ICI drugs			
Nivolumab	4	4	>0.99
Pembrolizumab	2	0	

NLR, neutrophil-lymphocyte ratio.
